# Synergistic Effects of Amikacin With Imipenem and Ciprofloxacin Against Multidrug‐Resistance Uropathogenic *Escherichia coli*

**DOI:** 10.1155/cjid/3283079

**Published:** 2026-06-23

**Authors:** Sahar Sadr Moharerpour, Fatemeh Ahmadi Parviji, Kasra Javadi, Mehrdad Halaji

**Affiliations:** ^1^ Non-Communicable Pediatric Diseases Research Center, Health Research Institute, Babol University of Medical Sciences, Babol, Iran, mubabol.ac.ir; ^2^ Student Research Committee, Babol University of Medical Sciences, Babol, Iran, mubabol.ac.ir; ^3^ Infectious Diseases and Tropical Medicine Research Center, Health Research Institute, Babol University of Medical Sciences, Babol, Iran, mubabol.ac.ir; ^4^ Department of Laboratory Sciences, School of Allied Medical Sciences, Babol University of Medical Sciences, Babol, Iran, mubabol.ac.ir

**Keywords:** drug synergy (synergism), *Escherichia coli*, minimum inhibitory concentration (MIC), multidrug-resistant (MDR)

## Abstract

**Introduction:**

There are limited therapeutic options available for treating aminoglycoside‐ and carbapenem‐resistant Gram‐negative bacteria. Therefore, there is an urgent need for novel effective antimicrobial agents. Numerous studies have reported the enhanced efficacy of antibiotic combinations against multidrug‐resistant (MDR) strains compared to monotherapy. This study aimed to investigate the potential synergistic effects of amikacin in combination with imipenem and ciprofloxacin against clinical isolates of MDR *Escherichia coli* to identify the most promising antibiotic combinations for treating infections caused by these isolates.

**Methods:**

In this experimental study, 20 MDR *E. coli* isolates were examined. The minimum inhibitory concentration (MIC) and checkerboard method were determined for amikacin, imipenem, and ciprofloxacin.

**Results:**

MIC determination revealed ciprofloxacin MICs of 4–512 μg/mL, imipenem 0.125–32 μg/mL, and amikacin 1–128 μg/mL. Resistance rates were 55% (11/20) for amikacin, 100% (20/20) for ciprofloxacin, and 55% (11/20) for imipenem. Synergistic effects were observed between amikacin and imipenem in 50% of isolates (10/20) and between amikacin and ciprofloxacin in 40% (8/20), with no antagonistic interactions detected.

**Conclusions:**

The combination of amikacin with imipenem or ciprofloxacin resulted in synergistic and additive effects in some cases. These findings suggest that antibiotic combination therapy could serve as an effective strategy in managing infections caused by antibiotic‐resistant isolates.

## 1. Introduction

Urinary tract infections (UTIs) are among the most prevalent clinical infections globally, with an estimated 150 million cases reported annually, contributing to over $6 billion in direct healthcare costs [1]. *Escherichia coli* remains the predominant causative pathogen, accounting for 80%–90% of community‐acquired UTIs and 30%–50% of nosocomial UTIs. Despite medical advancements, recurrent UTIs affect approximately 25% of women within six months of an initial infection, posing a significant clinical challenge [2].

A key concern in managing UTIs in children is that a single episode may serve as an early indicator of an underlying renal abnormality [3]. UTIs are the initial presentation in approximately 30% of children with congenital anomalies of the kidney and urinary tract (CAKUT) [3, 4]. As a result, since the 1960s, pediatric UTI management has been guided by the belief that recurrent infections, especially in the presence of vesicoureteral reflux (VUR), significantly elevate the risk of developing chronic kidney disease (CKD), hypertension, and ultimately, end‐stage renal disease (ESRD) [5, 6].

The emergence of multidrug‐resistant (MDR) uropathogenic *E. coli* (UPEC) has become a critical concern, with widespread dissemination in hospital settings and increasing isolation from community‐acquired infections [7]. This alarming trend underscores the urgent need for novel antimicrobial agents. Studies from Europe and North America have documented high MDR prevalence among *E. coli* isolates, with even higher resistance rates reported in Asian and African countries [8–10].

Carbapenems, traditionally considered last‐resort antibiotics for drug‐resistant Gram‐negative infections, exhibit stability against most β‐lactamases due to their unique β‐lactam ring structure [11]. Similarly, aminoglycosides exert potent bactericidal effects by targeting bacterial ribosomes, thereby inhibiting protein synthesis, a mechanism particularly effective in systemic infections [12]. However, resistance to these agents, mediated by aminoglycoside‐modifying enzymes and 16S rRNA methyltransferases, is increasingly prevalent among carbapenem‐resistant isolates [13]. With limited therapeutic options available for carbapenem‐resistant Gram‐negative bacteria, the development of effective antimicrobial strategies remains a priority [14, 15].

Antibiotic combination therapy has emerged as a promising approach to combat MDR pathogens [16]. Studies demonstrate that synergistic combinations particularly aminoglycosides paired with *β*‐lactams, glycopeptides, or carbapenems can enhance efficacy by targeting bacterial cell walls and protein synthesis simultaneously [17, 18]. Fluoroquinolones, such as ciprofloxacin, are widely used for *E. coli* infections but face rising resistance due to multiple mechanisms, including target mutations and efflux pump overexpression [19, 20]. The escalating resistance to fluoroquinolones complicates treatment regimens and worsens clinical outcomes [21].

Recent data highlight a dramatic increase in MDR UPEC infections, with isolates showing reduced susceptibility to imipenem, ciprofloxacin, and cephalosporins [22]. Combination therapy offers potential advantages, including broadened antimicrobial spectra, additive/synergistic effects, and delayed resistance development [7, 23]. Consequently, this study evaluated the in vitro synergistic activity of amikacin combined with imipenem and ciprofloxacin against clinical MDR UPEC isolates, aiming to identify effective therapeutic alternatives.

## 2. Materials and Methods

### 2.1. Bacterial Isolation

This study utilized 150 confirmed UPEC isolates. Previously, these isolates were collected from patients hospitalized between 2022 and 2024 in hospitals affiliated with Babol University of Medical Sciences, located in northern Iran [24, 25]. From these, 20 isolates were selected for further testing based on confirmed MDR (resistance to ≥ 3 antibiotic classes) and/or extended‐spectrum β‐lactamase (ESBL) production (Table 1).

**TABLE 1:** Antibiotic resistance profiles of UPEC isolates.

Isolate number	ESBL	MDR	Antibiotic resistance pattern
13	+	+	CAZ, AMC, CTX, SAM, SXT, CP, OFX, NA, NOR
23	+	+	CAZ, AMC, CFM, CTX, SAM, CRO, SXT, CP, OFX, NA, NOR
33	+	+	CAZ, AMC, CFM, CTX, SAM, CRO, SXT, CP, OFX, NA, NOR
32	−	+	MEM, SAM, CRO, AN, GM, SXT, CP, OFX, NA, NOR
39	+	+	MEM, CAZ, AMC, CFM, CTX, SAM, CRO, AN, GM, SXT, CP, OFX, NA, NOR
62	+	+	CAZ, AMC, CFM, CTX, SAM, CRO, AN, GM, SXT, CP, OFX, NA, NOR
63	+	+	CAZ, AMC, CFM, CTX, SAM, CRO, AN, GM, SXT, CP, OFX, NA, NOR
70	+	+	MEM, CAZ, AMC, CFM, CTX, SAM, CRO, SXT, CP, OFX, NA, NOR
75	+	+	CAZ, CFM, CTX, CRO, GM, SXT, CP, OFX, NA, NOR
76	+	+	CAZ, CFM, CTX, CRO, GM, SXT, CP, OFX, NA, NOR
37	−	+	MEM, CTX, SAM, CRO, AN, GM, SXT, CP, OFX, NA, NOR
52	−	+	MEM, FM, CTX, SAM, CRO, AN, GM, SXT, CP, OFX, NA, NOR
55	−	+	MEM, FM, CAZ, CFM, CTX, SAM, CRO, AN, GM, SXT, CP, OFX, NA, NOR
57	+	+	CAZ, AMC, CTX, AN, GM, OFX, NA, NOR, CP
74	+	+	MEM, FM, CAZ, CFM, SAM, CRO, AN, GM, SXT, CP, OFX, NA, NOR
10	+	+	CAZ, AMC, CFM, CTX, SAM, CRO, AN, SXT, CP, OFX, NA, NOR
20	+	+	MEM, CAZ, CFM, CTX, SAM, AN, SXT, CP, NA
22	+	+	MEM, CAZ, CFM, CTX, SAM, AN, CP, NA, AMC
26	−	+	FM, CTX, SAM, AN, SXT, CP, NA, AMC
27	−	+	FM, SAM, SXT, CP

*Note:* CAZ: ceftazidime; CFM: cefixime; CTX: cefotaxime; SAM: ampicillin–sulbactam; CRO: ceftriaxone; SXT: trimethoprim‐sulfamethoxazole; CP: ciprofloxacin; OFX: ofloxacin; NOR: norfloxacin; MEM: meropenem; AN: amikacin; GM: gentamicin; FM: nitrofurantoin.

Abbreviations: AMC, amoxicillin–clavulanic acid; NA, nalidixic acid.

### 2.2. Antimicrobial Susceptibility Testing

The Kirby–Bauer disk diffusion method was employed to assess the susceptibility of UPEC isolates to a range of antibacterial agents, including amikacin, ciprofloxacin, norfloxacin, ofloxacin, and nalidixic acid. Testing was performed on Mueller–Hinton agar (MHA) using antibiotic disks provided by Padtan Teb Co., Iran. Interpretation of the results followed the Clinical and Laboratory Standards Institute (CLSI) 2024 guidelines [26]. *E. coli* ATCC 25922 was used as the quality control strain to ensure the reliability of the antimicrobial susceptibility testing.

### 2.3. Detection of ESBL‐Producing *E*. *coli*

ESBL production was assessed with the combined disk diffusion test in line with CLSI guidance [26]. In brief, ceftazidime (30 µg) and cefotaxime (30 µg) disks were applied alone and alongside clavulanic acid (10 µg). If the inhibition zone for either agent increased by ≥ 5 mm in the presence of clavulanate, relative to the drug tested alone, the isolate was interpreted as ESBL‐positive. Quality control strains were run with each grouping: *E*. *coli* ATCC 25922 (negative control) and *Klebsiella pneumoniae* ATCC 700603 (positive control).

### 2.4. Minimal Inhibitory Concentration (MIC)

The MIC values of the tested antimicrobial agents were determined using the broth microdilution method by the CLSI 2024 guidelines [26]. Serial two‐fold dilutions of amikacin, imipenem, and ciprofloxacin were prepared in 96‐well microtiter plates containing Mueller–Hinton broth (MHB). The concentration ranges tested were 512–1 µg/mL for amikacin, 64–0.0625 µg/mL for imipenem, and 1024–1 µg/mL for ciprofloxacin. After inoculation, plates were incubated at 37°C for 20–24 h. Bacterial growth was monitored by measuring the optical density at 600 nm using a microplate reader (Bio‐Rad, USA). Wells containing only MHB and bacterial inoculum served as growth controls and were considered to represent 100% growth. The extent of growth inhibition in each well was calculated relative to this control. The MIC was defined as the lowest concentration of the antibiotic that completely inhibited visible bacterial growth after 24 h of incubation at 37°C.

### 2.5. Checkerboard Assay

The checkerboard assay evaluated the potential synergistic interaction between amikacin and either imipenem or ciprofloxacin [27]. In accordance with CLSI guidelines, serial two‐fold dilutions of the antibiotics were prepared to at least twice their respective MIC values. These dilutions were prepared before testing and combined in 96‐well microtiter plates containing various amikacin concentrations in MHB. Each well was inoculated with 100 μL of a bacterial suspension adjusted to 5 × 10^5^ CFU/mL and incubated at 37°C for 24 h.

To assess interactions, the fractional inhibitory concentration (FIC) index was calculated as follows:

FIC index = FIC_A + FIC_B = (MIC of Drug A in combination/MIC of Drug A alone) + (MIC of Drug B in combination/MIC of Drug B alone).

The results were interpreted as follows: synergy (FIC index ≤ 0.5), additive effect (> 0.5 to ≤ 1), indifference (> 1 to ≤ 2), and antagonism (> 2) [28].

### 2.6. Statistical Analysis

Statistical analysis was conducted using SPSS (Version 27.0; IBM Corp., Armonk, NY, USA). Given that MIC values are derived from discrete two‐fold dilutions, they were reported as medians with ranges. MIC50 and MIC90 were calculated as the 50th and 90th percentiles of the isolate‐level MIC distributions. Paired comparisons of isolate MICs obtained under monotherapy versus combination testing were performed using the two‐sided Wilcoxon signed‐rank test, with *p* < 0.05 considered statistically significant. Checkerboard assay outcomes were reported as the number and percentage of isolates exhibiting synergistic, additive, indifferent, or antagonistic interactions, and 95% confidence intervals (CIs) for proportions were calculated using the Wilson score method.

## 3. Results

### 3.1. Antibiotic Resistance Pattern

The majority of isolates demonstrated resistance to fluoroquinolone and quinolone antibiotics, including ciprofloxacin, norfloxacin, ofloxacin, and nalidixic acid. Table 1 presents the antibiotic resistance profiles and the production of ESBL enzymes.

### 3.2. Determination of MIC for Amikacin, Imipenem, and Ciprofloxacin

The isolate‐level MICs are presented in Table 2. Ciprofloxacin MICs ranged from 4 to 512 µg/mL (median, 64 µg/mL), imipenem MICs ranged from 0.125 to 32 µg/mL (median, 8 µg/mL), and amikacin MICs ranged from 1 to 128 µg/mL (median, 16 µg/mL). The MIC50/MIC90 values were 64/512 µg/mL for ciprofloxacin, 8/16 µg/mL for imipenem, and 16/64 µg/mL for amikacin. Based on CLSI 2024 breakpoints for amikacin (*S* ≤ 4 µg/mL, I = 8 µg/mL, R ≥ 16 µg/mL), 55% (11/20) of isolates were resistant, 20% (4/20) were intermediate, and 25% (5/20) were susceptible. Similarly, 55% (11/20), 30% (6/20), and 15% (3/20) of isolates were resistant, susceptible, and intermediate to imipenem, respectively. All isolates were resistant to ciprofloxacin (Table 2).

**TABLE 2:** Minimum inhibitory concentration (MIC) of amikacin, ciprofloxacin, and imipenem for UPEC isolates.

Clinical isolate number	MIC ciprofloxacin (µg/mL)	MIC imipenem (µg/mL)	MIC amikacin (µg/mL)
37	128	32	4
74	64	16	4
52	128	16	64
55	256	16	128
20	64	1	4
22	64	1	32
26	4	1	16
27	32	1	8
23	64	2	16
39	256	16	128
57	64	2	8
62	512	16	8
63	128	8	8
70	512	16	16
76	128	16	64
75	512	8	16
10	32	0.125	4
13	64	32	16
32	8	0.125	1
33	64	2	16
MIC50	64	8	16
MIC90	512	16	64

Abbreviation: MIC, minimum inhibitory concentration.

### 3.3. Evaluation of the Combined Effect of Amikacin and Imipenem

The antibacterial interaction between amikacin and imipenem was assessed using the checkerboard assay (Table 3). Synergistic interactions were observed in 50.0% of isolates (10/20; 95% CI, 29.9–70.1). Additionally, 35.0% (7/20) exhibited an additive effect and 15.0% (3/20) showed indifference. Notably, no antagonistic interactions were detected (0/20; 95% CI, 0.0–16.1). Compared with MICs obtained under monotherapy, combination testing significantly reduced MICs for both imipenem (median reduction, 2.0 two‐fold dilutions; range, 0–5; Wilcoxon *p* < 0.001) and amikacin (median reduction, 2.0 two‐fold dilutions; range, 0–3; Wilcoxon *p* < 0.001).

**TABLE 3:** Checkerboard assay results (MIC and effect type) for amikacin with imipenem.

Isolate	Amikacin (A) (µg/mL)	Imipenem (B) (µg/mL)	(A + B)	(B + A)	FICI	Effect type
37	4	32	2	16	1	Additive
74	4	16	2	2	0.625	Additive
52	64	16	16	2	0.375	Synergism
55	128	16	32	4	0.5	Synergism
20	4	1	2	0.125	0.625	Additive
22	32	1	8	0.25	0.5	Synergism
26	16	1	16	0.125	1.125	Indifference
27	8	1	2	0.25	0.5	Synergism
23	16	2	8	0.0625	0.53125	Additive
39	128	16	64	16	1.5	Indifference
57	8	2	2	0.5	0.5	Synergism
62	8	16	8	1	1.0625	Indifference
63	8	8	2	2	0.5	Synergism
70	16	16	4	1	0.3125	Synergism
76	64	16	16	2	0.375	Synergism
75	16	8	8	4	1	Additive
10	4	0.125	0.5	0.0625	0.625	Additive
13	16	32	4	8	0.5	Synergism
32	1	0.125	0.5	0.0625	1	Additive
33	16	2	4	0.5	0.5	Synergism

Abbreviation: FICI, Fractional Inhibitory Concentration Index.

### 3.4. Evaluation of the Combined Effect of Amikacin and Ciprofloxacin

The interaction between amikacin and ciprofloxacin was evaluated using the checkerboard assay (Table 4). Synergy was detected in 40.0% of isolates (8/20; 95% CI, 21.9–61.3), while an additive effect was observed in another 40.0% (8/20). The remaining 20.0% (4/20) were classified as indifferent. No antagonistic interactions were detected (0/20; 95% CI, 0.0–16.1). Combination testing significantly reduced ciprofloxacin MICs (median reduction, 2.5 two‐fold dilutions; range, 0–5; Wilcoxon *p* < 0.001) and amikacin MICs (median reduction, 1.5 two‐fold dilutions; range, 0–5; Wilcoxon *p* < 0.001) relative to monotherapy.

**TABLE 4:** Checkerboard assay results (MIC and effect type) for amikacin with ciprofloxacin.

Isolate	Amikacin (A) (µg/mL)	Ciprofloxacin (B) (µg/mL)	(A + B)	(B + A)	FICI	Effect type
37	4	128	1	128	1.25	Indifference
74	4	64	2	16	0.75	Additive
52	64	128	16	32	0.5	Synergism
55	128	256	32	128	0.75	Additive
20	4	64	0.5	8	0.25	Synergism
22	32	64	16	32	1	Additive
26	16	4	16	0.5	1.125	Indifference
27	8	32	2	4	0.375	Synergism
23	16	64	4	16	0.5	Synergism
39	128	256	64	32	0.625	Additive
57	8	64	4	8	0.625	Additive
62	8	512	8	64	1.125	Indifference
63	8	128	8	8	1.0625	Indifference
70	16	512	8	32	0.5625	Additive
76	64	128	16	16	0.375	Synergism
75	16	512	8	256	1	Additive
10	4	32	0.125	1	0.0625	Synergism
13	16	64	8	16	0.75	Additive
32	1	8	0.25	2	0.5	Synergism
33	16	64	4	16	0.5	Synergism

Abbreviation: FICI, Fractional Inhibitory Concentration Index.

## 4. Discussion

In this study, the antibiotic resistance patterns and the synergistic effects of antibiotic combinations were evaluated in 20 selected UPEC isolates. These isolates, chosen from a pool of 150 confirmed strains, demonstrated resistance to fluoroquinolones (ciprofloxacin, norfloxacin, ofloxacin, and nalidixic acid) and/or amikacin. These findings highlight the high prevalence of MDR among UPEC isolates, aligning with global trends of increasing antibiotic resistance in Gram‐negative bacteria, particularly in clinical environments.

All 20 isolates were resistant to ciprofloxacin, while resistance to amikacin and imipenem was observed in 55% of the isolates. The high resistance level to fluoroquinolones may be attributed to their widespread use in treating UTIs in the studied region, exerting selective pressure that fosters the emergence of resistant strains. Moreover, the production of ESBL, as reported in Table 1, can be considered a major mechanism of resistance to beta‐lactam antibiotics and potentially contribute to reduced susceptibility to other antibiotic classes, such as fluoroquinolones. Recent studies, including that by Patel et al. (2023), have similarly reported that ESBL‐producing UPEC strains often exhibit multidrug resistance to fluoroquinolones and aminoglycosides, supporting our findings.

Based on MIC results, resistance to ciprofloxacin ranged from 4 to 512 µg/mL, indicating a high resistance level among isolates, with both MIC_50_ and MIC_90_ showing elevated values. In contrast, imipenem, with a MIC range of 0.125 to 32 µg/mL and 30% sensitivity among isolates, remains a comparatively effective treatment option. Farhan et al. (2023) also reported high resistance rates to amikacin and imipenem among MDR *E*. *coli* isolates, consistent with our findings [29].

One of the main findings of this study was the synergistic/additive activity of amikacin combinations. The combination of amikacin and ciprofloxacin exhibited synergy in 40% of isolates and additive effects in 40%, with no antagonism observed; however, high ciprofloxacin MICs may limit its clinical utility. Amikacin–imipenem demonstrated synergy in 50% of isolates (additive 35%, indifferent 15%), suggesting higher therapeutic potential due to complementary mechanisms of action. Drago et al. (2005) reported synergistic and additive effects for levofloxacin combined with imipenem or amikacin against ESBL‐producing *E*. *coli* [30]. Loho et al. (2018) demonstrated that doripenem combined with amikacin significantly reduced MICs across various strains, although synergy effects were more limited [31]. Yang et al. (2023) showed that meropenem plus amikacin dramatically reduced MICs below susceptibility breakpoints and gave FICI < 0.5 against MDR *P*. *aeruginosa*, indicating strong synergism [32]. Moreover, Bayatinejad et al. (2023) reported synergistic activity between colistin and amikacin in 70% of carbapenem‐resistant *K*. *pneumoniae* [33], highlighting another potent aminoglycoside combination.

The study by Terbtothakun et al. (2021) analyzed antibiotic resistance in 19 carbapenem‐resistant *E. coli* (CREC) isolates, all of which were resistant to imipenem, with MIC50 and MIC90 values of 64 and 128 µg/mL, respectively. In contrast, amikacin showed high efficacy against 14 isolates, indicating relative susceptibility to aminoglycosides. Their study also assessed the synergistic effects of meropenem with various aminoglycosides, finding synergy in 84% of isolates with meropenem–gentamicin and streptomycin, 79% with meropenem–kanamycin and tobramycin, and 68% with meropenem–amikacin [34]. These results underscore the potential of antibiotic combinations as effective interventions against carbapenem‐resistant isolates.

Carbapenem–aminoglycoside combinations, such as meropenem and amikacin, target both cell wall synthesis and protein synthesis. Carbapenems may enhance the uptake of aminoglycosides by disrupting the peptidoglycan layer, while aminoglycosides contribute to bacterial membrane damage [32]. Although carbapenems and aminoglycosides are generally effective against Gram‐negative infections, reliance on monotherapy may promote resistance and lead to adverse effects. Therefore, combination therapy remains an important strategy for managing MDR *E*. *coli* infections [35–37].

The findings of this study suggest that the combination of amikacin and imipenem is an effective therapeutic option for managing severe infections caused by MDR *E. coli* strains. This combination not only demonstrated strong synergistic activity but also has the potential to reduce the risks associated with monotherapy and alleviate clinical treatment burdens.

### 4.1. Limitations

The main limitations of this study include the small number of selected isolates, the in vitro design, the inclusion of only urinary isolates, and the evaluation of only two antibiotic combinations. Future studies should include a larger and more diverse collection of isolates from different clinical sources, as well as in vivo and clinical evaluations.

## 5. Conclusion

The evaluation of MIC values for amikacin, imipenem, and ciprofloxacin showed a wide range of resistance among isolates. Notably, all isolates were resistant to ciprofloxacin, while resistance to amikacin and imipenem was observed in 55% of cases. The checkerboard analysis of antibiotic combinations revealed that the use of amikacin with imipenem or ciprofloxacin led to synergistic or additive effects in several instances. These results suggest that antibiotic combinations may serve as an effective strategy against infections caused by resistant isolates.

## Author Contributions

Sahar Sadr Moharerpour: conceptualization, methodology, and writing–original draft. Fatemeh Ahmadi Parviji: methodology, formal analysis, data curation, and writing–original draft. Kasra Javadi: conceptualization, methodology, and writing–review and editing. Mehrdad Halaji: project administration, conceptualization, methodology, validation, and writing–review and editing.

## Funding

This study, part of Fatemeh Ahmadi Parviji’s MD thesis, was supported by Babol University of Medical Sciences in Babol, I.R., Iran (grant number: 724135557).

## Disclosure

All authors have read and approved the final manuscript.

## Ethics Statement

The research was approved by the Research Ethics Committee of Babol University of Medical Sciences (IR.MUBABOL.HRI.REC.1402.268), Babol, Iran. These patients routinely referred to the microbiology laboratory, and a urine sample was taken and then we only used bacteria isolates obtained from urine culture. A written informed consent was obtained from all participants. In this study, we did not use samples from minors. The study was performed in accordance with the Declaration of Helsinki. The study used bacteria isolated from clinical samples in the clinical microbiology laboratory.

## Conflicts of Interest

The authors declare no conflicts of interest.

## Data Availability

The data that support the findings of this study are available from the corresponding author upon reasonable request.
